# Avoiding Missed Diagnoses of Pharyngeal Foreign Bodies in Postmortem Investigations: A Case Report of Denture-Related Asphyxia

**DOI:** 10.7759/cureus.95028

**Published:** 2025-10-21

**Authors:** Ikuto Takeuchi, Motoo Yoshimiya, Atsushi Ueda, Yu Kakimoto

**Affiliations:** 1 Department of Forensic Medicine, Tokai University School of Medicine, Isehara, JPN

**Keywords:** acrylic resin denture, asphyxia, external postmortem examination, pharyngeal foreign body, postmortem computed tomography

## Abstract

Foreign body asphyxia due to pharyngeal denture impaction is an uncommon but potentially fatal event, especially in elderly individuals. This report describes a forensic case in which an acrylic resin denture was discovered impacted in the laryngopharynx during postmortem examination. A faint metallic artifact detected on postmortem computed tomography (PMCT) raised suspicion of a foreign body, prompting laryngeal inspection, which revealed the missing lower denture lodged in the laryngopharynx. The denture was composed mainly of radiolucent acrylic resin, with only a minimal metallic component, which made its detection difficult on imaging studies. No other fatal findings were identified, and the cause of death was most consistent with asphyxia due to airway obstruction. This case highlights the diagnostic value of combining PMCT with direct laryngeal inspection, especially when imaging is inconclusive. It also underscores the importance of recognizing subtle imaging signs and accounting for missing prosthetic devices during postmortem evaluations, particularly in elderly individuals. In countries like Japan, where postmortem assessments may rely solely on external examination and PMCT, this case emphasizes the need for vigilance regarding airway obstruction even in the absence of an autopsy.

## Introduction

Acrylic resin dentures are widely used to support masticatory and speech functions in older adults, contributing significantly to oral health-related quality of life [1]. However, accidental ingestion or aspiration of dentures is a known risk, particularly among elderly individuals or those with cognitive impairments [2]. There have been multiple reports of dentures causing gastrointestinal foreign bodies or becoming impacted in the pharynx or larynx [3-5]. Since acrylic materials are radiolucent, they may be difficult to detect on CT or X-ray, potentially leading to diagnostic delays or oversights [3,4].

In Japan, when the cause of death is uncertain, a physician conducts an external postmortem examination before deciding whether an autopsy is warranted [6]. When there are no obvious signs of trauma or criminal activity, cause-of-death assessments are sometimes made based solely on external inspection and postmortem computed tomography (PMCT), without an autopsy. In some settings, limitations in imaging capabilities or examination techniques may hinder the detection of foreign bodies.

This report presents a forensic case in which a faint metallic artifact on PMCT prompted a laryngeal inspection, revealing pharyngeal impaction of a resin denture. We discuss practical strategies to avoid overlooking airway obstruction due to foreign bodies, based on insights from this case.

## Case presentation

An elderly man in his 80s was found lying supine in his living room by family members. Rigor mortis and signs of decomposition were already present, and no resuscitative measures were attempted. A shopping receipt dated five days prior was found at the scene, suggesting that the decedent had been deceased for several days before discovery, which explains the advanced decomposition changes observed on examination. During the external postmortem examination conducted by the police, an upper denture was found in the oral cavity, but the lower denture was missing. The decedent had been living alone and had a medical history of complete denture use, cervical spinal tumor, and prostate cancer. A shopping receipt dated five days prior was discovered at the scene, presumed to be the last record of activity.

**Figure 1:**
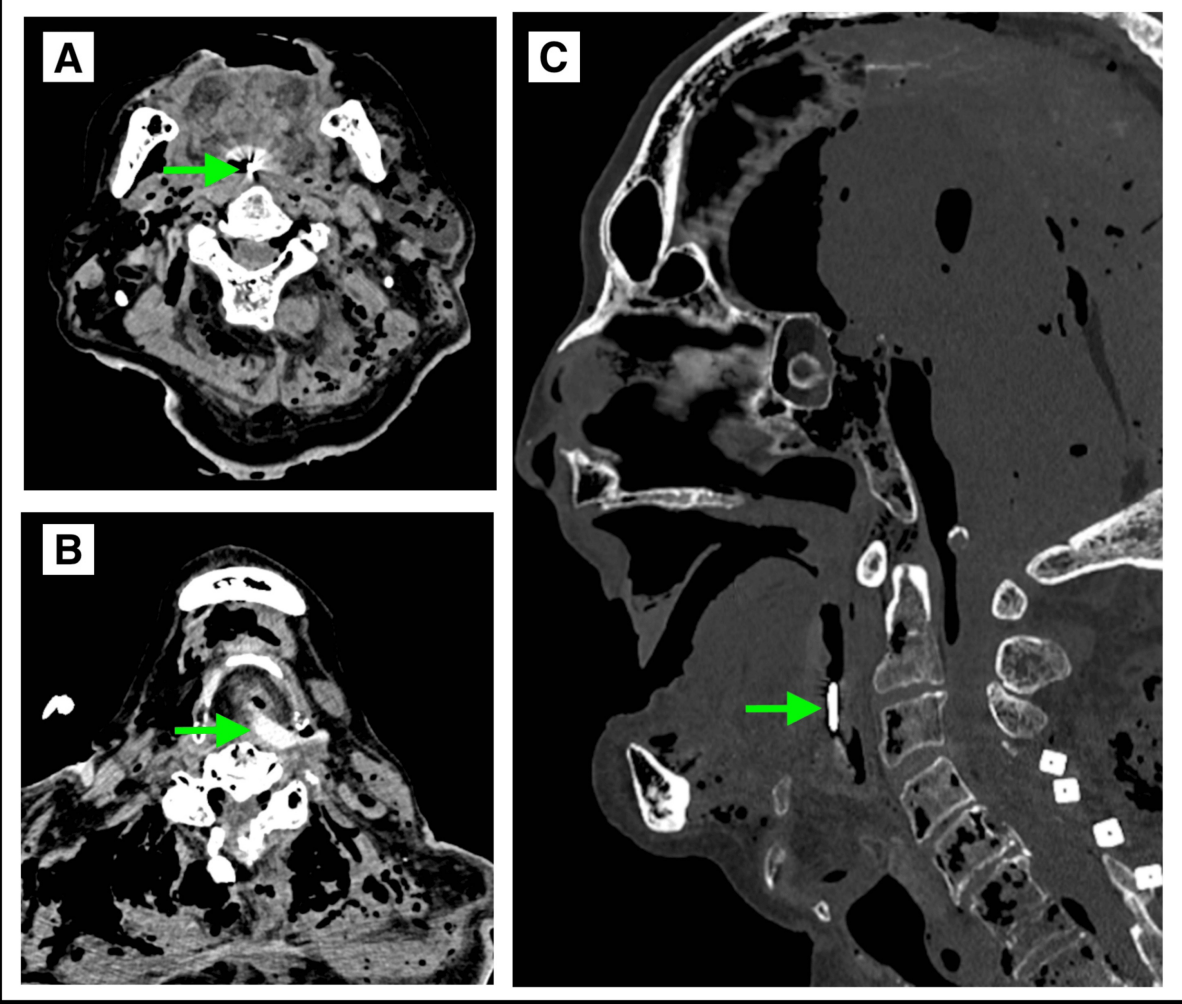
Postmortem CT findings (A) Axial image at the level of the second cervical vertebra (C2) showing a punctate hyperdense area with imaging artifact (green arrow). (B) Axial image at the level of the fourth cervical vertebra (C4) demonstrating a hyperdense area (green arrow) that is denser than the surrounding soft tissue. (C) Sagittal image showing a linear hyperdense structure in the pharyngeal region (green arrow).

A postmortem examination was performed at a university forensic medicine department the following day. The body showed extensive decomposition, including marbling, skin blistering and rupture, and parchment-like changes, without apparent trauma or lethal pathology. Due to the advanced decomposition, typical external or internal signs of asphyxia, such as facial congestion, cyanosis, petechiae, or pulmonary edema, could not be reliably evaluated. No distinct traumatic or hemorrhagic findings were observed. Although advanced decomposition limited detailed interpretation on PMCT, no large hematoma, major thoracic or abdominal bleeding, or fractures suggestive of fatal trauma were identified. The heart and great vessels were markedly decomposed, and no evidence of rupture or hemopericardium was seen. While subtle pathological changes such as myocardial infarction could not be evaluated under these conditions, there were no radiological findings suggesting massive hemorrhage or violent injury. In addition, imaging revealed a faint linear hyperdense area in the laryngopharynx (Figures 1A-1C). 

**Figure 2:**
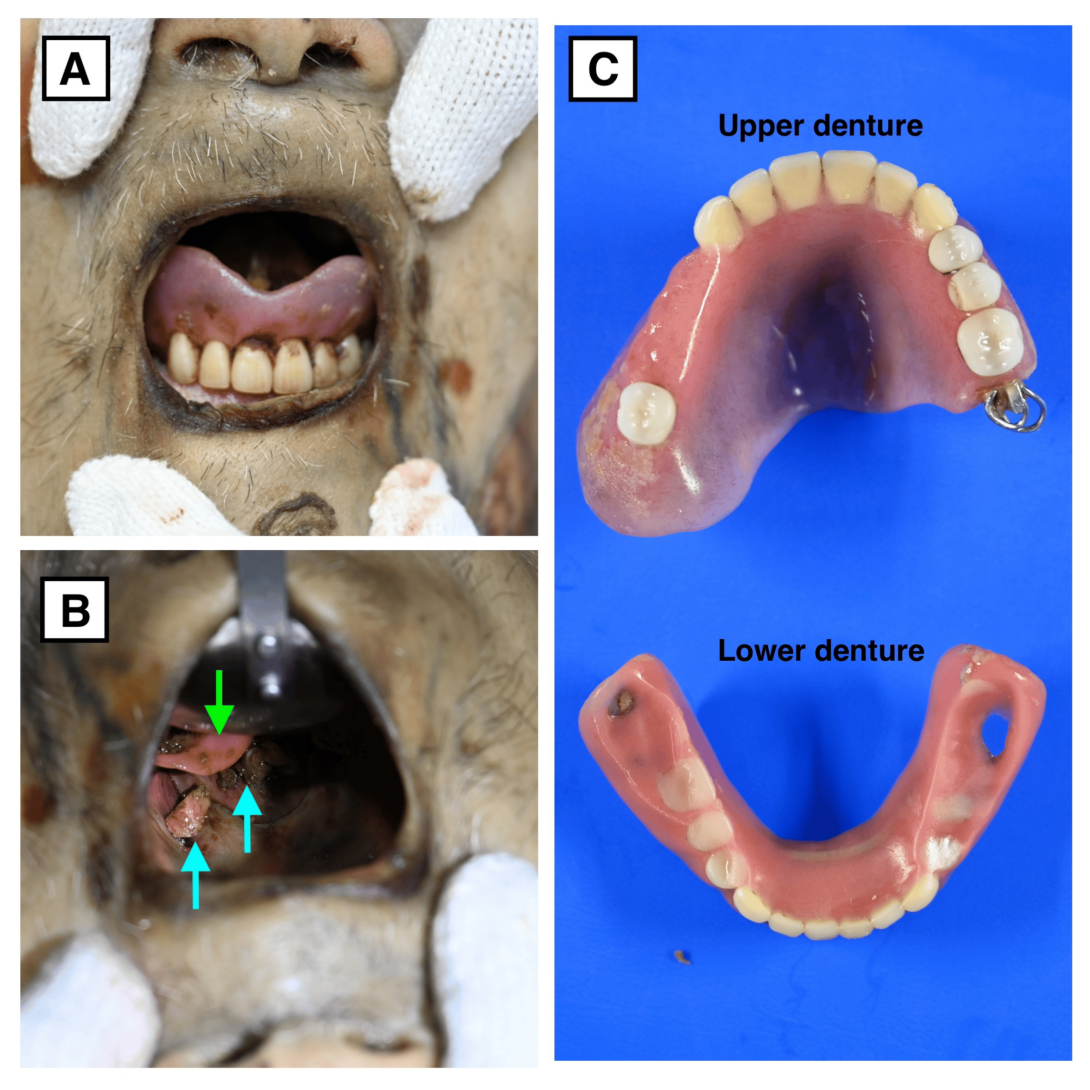
Denture-related findings. (A) Photograph showing the upper complete denture remaining in the oral cavity. (B) Inspection through the oral cavity revealing the impacted denture in the laryngopharynx. The green arrow indicates part of the denture, and the blue arrow indicates food residue. (C) Photograph showing both dentures: the upper denture positioned in the upper part of the image and the impacted lower denture in the lower part.

Based on this finding and the absence of the lower denture (Figure 2A), an examination of the oral cavity and larynx was conducted. 

This revealed a denture impacted along its long axis in the laryngopharynx, accompanied by a small amount of food residue (Figure 2B). Considerable force was needed for removal, indicating firm impaction.

The extracted artificial denture was primarily composed of acrylic resin with a small metallic component and was designed to fit a single remaining tooth (Figure 2C). Both the metallic portion and the surrounding resin were visible on CT. No other foreign bodies or fatal lesions were identified, and the police investigation found no evidence of foul play or suspicious circumstances at the scene. The cause of death was determined to be asphyxia due to airway obstruction by the denture.

## Discussion

This case illustrates the value of combining imaging and direct observation in forensic death investigations. A faint metallic artifact on PMCT led to the discovery of a partially metallic acrylic resin denture impacted in the laryngopharynx, resulting in a diagnosis of asphyxia due to airway obstruction. While dentures serve essential functions in elderly populations, their accidental ingestion poses a serious risk [1,2]. In clinical settings, aspiration may be recognized during emergency care, but in postmortem contexts, delayed discovery can hinder identification of the cause of death. In this case, the foreign body was not recognized during the initial scene investigation. Materials like polymethyl methacrylate (PMMA), commonly used in dentures, are highly radiolucent and can be difficult to distinguish from soft tissues on CT or X-ray [7]. When little to no metal is present, dentures may not appear on imaging, with small metallic clasps sometimes being the only visible clues, as in this case [7]. The interpretation of such subtle imaging findings requires both experience and vigilance on the part of health professionals involved in postmortem imaging interpretation. When most of a denture is radiolucent, relying solely on imaging is insufficient. The pharyngeal and laryngeal regions, especially the piriform recess and glottic level, are anatomically predisposed to foreign body impaction, and careful evaluation of these areas is essential during imaging interpretation [7].

In postmortem CT evaluations, using structured worksheets has been reported to improve diagnostic accuracy. However, when it comes to the neck, these worksheets tend to focus primarily on evaluating cervical spine injuries [8]. As is known from the concept of inattentional bias, the tendency to overlook unexpected findings when attention is focused elsewhere, airway foreign bodies may go unnoticed unless specific attention is paid [9]. In this case, the faint linear hyperdense area observed in the laryngopharynx measured approximately 130 Hounsfield units (HU) on PMCT, which is consistent with the expected attenuation range of artificial acrylic resin materials. In general, acrylic resin shows CT values around 70 HU, whereas the surrounding soft tissues measure approximately 50 HU [10]. Although the value observed in this case was higher than those reported in previous studies, acrylic resin is known to have higher attenuation than soft tissue, and the observed difference was sufficient to allow distinction from the surrounding structures.

Even when imaging is inconclusive, direct observation techniques such as laryngeal inspection can contribute significantly to cause-of-death determination. In environments lacking PMCT, this method becomes even more critical. Previous reports have described cases where dentures were identified only through endoscopic or surgical procedures when not visible on imaging [3-5]. In this case, sudden cardiac death was also considered. However, PMCT, although limited by decomposition, showed no evidence of major hemorrhage, cardiac rupture, or aortic catastrophe, and there were no scene findings suggestive of sudden collapse from natural disease. While microscopic cardiac pathology or toxic causes cannot be excluded without histology and toxicology, the firmly impacted denture across the airway, with adherent food residue and positional compatibility with complete obstruction, provides the most direct explanation for the fatal event. Detailed autopsy, histological, and toxicological examinations were not performed because only external examination, PMCT, and laryngeal inspection were authorized. In addition, decomposition limited the radiological evaluation, especially of the heart and lungs. Therefore, microscopic cardiac or toxic causes cannot be ruled out, and the cause of death is described as “most consistent with asphyxia due to airway obstruction by a pharyngeally impacted denture.”

In Japan, the procedure for postmortem external examinations is not standardized nationwide. The Japanese Society of Legal Medicine has published the Manual for Postmortem External Examination (Version 7.0.5, 2025) as a reference, but it does not define laryngeal inspection as a routine component [11]. This may be partly because rigor mortis often limits the feasibility of laryngeal inspection during external examinations, especially when decomposition has already begun. However, endotracheal intubation using a laryngoscope is considered a basic skill for physicians, and laryngeal inspection to evaluate for foreign bodies or airway obstruction is a feasible practice in postmortem settings [12]. In an aging society like Japan, where elderly individuals face an increasing risk of aspiration due to declining swallowing function, laryngeal inspection should be considered when the cause of death is unclear [7,13]. Even in decomposed cases, dentures as artificial objects persist, and combining imaging with physical inspection can lead to accurate diagnoses. In this case, information from the scene, specifically the absence of the lower denture, was also key. Denture status is sometimes overlooked during postmortem examinations, but recognizing "what is missing" can be as critical as finding abnormalities. Laryngeal inspection in response to the missing denture enabled the direct identification of a pharyngeally impacted denture and, ultimately, the correct determination of the cause of death.

This case provides several practical lessons. First, attention to pharyngeal foreign bodies is essential during postmortem CT interpretation, as subtle findings can easily be overlooked. In addition, recognizing the absence of prosthetics such as dentures can serve as a critical indirect clue that prompts further investigation. Finally, diagnostic accuracy is greatly enhanced when imaging is complemented by direct inspection, underscoring the importance of combining both approaches in forensic practice. These perspectives are particularly important in forensic practice in aging populations, where they may help prevent the oversight of fatal airway obstructions. These points are especially important in countries like Japan, where doctors are sometimes asked to decide the cause of death based only on an external examination and postmortem CT. In such cases, it is important to always keep the possibility of airway obstruction in mind. Even if an autopsy is not done, careful observation and attention to missing dentures or unusual findings on CT can help avoid missing the true cause of death.

## Conclusions

This case highlights the successful use of postmortem computed tomography (PMCT) and oral and laryngeal inspection in identifying an acrylic resin denture impacted on the laryngopharynx, supporting a diagnosis most consistent with asphyxia due to airway obstruction. When imaging does not provide clear evidence, the absence of expected findings, such as a missing denture, may serve as a critical diagnostic clue that prompts further investigation.

In Japan and other countries where cause-of-death decisions are sometimes made based only on external postmortem examination and PMCT, this case emphasizes the importance of maintaining suspicion for airway obstruction by foreign bodies. To improve diagnostic accuracy in such cases, it is helpful to understand the limitations of imaging, consider the material properties of prosthetic devices, and perform direct inspection when necessary. These approaches may support more accurate forensic evaluations, particularly when an autopsy is not performed.
